# The physicochemical and environmental factors affecting the distribution of *Anopheles merus* along the Kenyan coast

**DOI:** 10.1186/s13071-015-0819-0

**Published:** 2015-04-11

**Authors:** Pamela C Kipyab, Battan M Khaemba, Joseph M Mwangangi, Charles M Mbogo

**Affiliations:** Kenya Medical Research Institute – Centre for Geographic Medicine Research-Coast, P.O Box 428, 80108 Kilifi, Kenya; Malaria Public Health Department, KEMRI-Wellcome Trust Research Programme, P.O. Box 43640, 00100 Nairobi, Kenya; Moi University, P.O Box 3900, 30100 Eldoret, Kenya

**Keywords:** *An*, *gambiae*, *An. merus*, Breeding habitats, Physicochemical parameters, Environmental parameters

## Abstract

**Background:**

Members of the *Anopheles gambiae* complex are the main transmitters of malaria. *Anopheles merus* is a member of the complex found along the Kenyan coast because it breeds in saline waters. An entomological study was conducted in Garithe Malindi District, to investigate the physicochemical and environmental factors affecting the distribution of *An. merus.*

**Methods:**

Field and laboratory studies were used to investigate the breeding habitats of the subspecies. Mosquito larvae were sampled using standard dipping technique from small pockets of pools, ponds, hoof prints, road drain, wells and mangrove swamps found in Garithe. All 3^rd^ and 4^th^ instars of *Anopheles* larvae sampled were identified microscopically into species. A representative of *Anopheles gambiae* complex was then identified to specific sibling species using r-DNA PCR technique.

The habitats were characterized based on temperature, conductivity, salinity, dissolved oxygen, total dissolved solids, pH, size, distance to nearest house, canopy coverage, surface debris, presence of algae, emergent plants, turbidity and habitat types.

**Results:**

A total of 159 morphologically identified late stage instar *Anopheles gambiae s.l* larvae were selected for r-DNA analysis by PCR. Out of these, 60.4% (n = 96) were *Anopheles merus,* 8.8% (n = 14) were *Anopheles arabiensis*, 18.2% (n = 29) were *Anopheles gambiae s.s* and 12.6% (n = 20) were unknown.

Using paired *t*-test (t _(121)_ = −3.331, P = 0.001) a significantly high proportion of *An. merus* was observed in all habitats compared to *An. arabiensis*, and *An. gambiae s. s*.

In habitat characterization, Pearson’s correlation analysis test showed different parameters being associated with the occurrence of *An. merus* larvae in the different habitats sampled. Six out of the 55 correlation coefficients (10.9%) were statistically significant, suggesting non-random association between some pairs of variables. Those that had a significantly high positive correlation with *An. merus* included temperature, salinity, conductivity, total dissolved solids and algae.

**Conclusions:**

Different physicochemical parameters and environmental parameters affect the occurrence of *An. merus*. In this study, higher temperatures accelerate the growth of the larvae and aids in growth of micro-organisms and algae which are food sources for the larvae. Saline waters favour the growth and development of *An. merus* larvae; they are also able to develop in a range of saline waters. Conductivity, total dissolved solids and canopy coverage are among the important factors influencing the development and abundance of *An. merus* larvae in their habitats. Habitat type also influences the abundance of *An. merus* larvae. They mainly prefer to breed in pools and ponds, but not swamps, hoof prints and wells.

## Background

The *Anopheles gambiae* complex is primarily responsible for approximately 80% of global malaria morbidity and mortality that occurs in sub-Saharan Africa [1]. *An. merus* is a member of the *An. gambiae* complex. In East Africa, *An. merus* is sometimes considered to play a secondary role in malaria transmission [2]. However emerging evidence gathered in recent studies in Madagascar and coastal parts of Tanzania implicates *An. merus* as a primary transmitter of malaria [3,4].

*An. merus* is known to breed in salty waters [5,6]. Studies on *An. merus* in Jimbo village on the Kenyan coast, observed that peak larval densities of *An. merus* occurred in waters with a salinity range of 30-50%. The larvae were also capable of completing development in alkaline saline water collected from an inland location around Lake Jipe in Kenya [5]. Studies in Madagascar also observed that *An. merus* larvae bred in crab-holes in the Betsiboka estuary with salinity of 0.07%. Larval mosquitoes were also collected in water which contained ions of Na^+^ 13, K^+^ 0.3, Ca^++^ 0.62, and Mg^++^ 1.1 [3].

Laboratory experiments done on the larvae of *An. merus* showed that at 24°C, survival rates from egg to fourth instar larvae showed a significantly better survival rate of 46.4% in 25% salinity compared with 15.5% freshwater [7].

Previous studies on *An. gambiae s.l* larvae showed that small habitats were more productive for Anopheline mosquitoes compared to large larval habitats during the rainy season [8]. This is because, larval predation is less prevalent in temporary habitats than it is in large permanent habitats [9,10], and again, open habitats tend to produce more algae which is the main food source for *An. gambiae s.l*, than shaded habitats [11]. *An. gambiae s. l* may have evolved to exploit these favourable conditions by selecting small and open habitats for oviposition. Stream pools and puddles are shallow and tend to be having lower complexity in terms of debris and vegetation cover. This means that the larval development tends to be faster due to higher temperatures and density remains high due to lower predator risk. The swamps, which are big in size, have higher complexity, which results in higher concentration of other invertebrate species [12], which could be important as predators or competitors for *Anopheles* larvae.

Knowledge of the preferred habitat of a mosquito is critical when considering the management of the vector species [11]. Mosquitoes commute between blood-meal hosts and breeding site. Thus, heterogeneity in human biting reflects underlying spatial heterogeneity in the distribution and suitability of larval habitat as well as inherent differences in the attractiveness, suitability and distribution of blood-meal hosts. One of the possible strategies of malaria control is to identify local vector species and then attack water bodies that contain their larvae [13].

With regard to transmission reduction, attention must be paid to the areas of greatest vector abundance [14,15]. Besides geographic location, knowledge of ecological features of mosquito breeding sites is a potential key element for implementing efficient and effective larvae control measures. Such measures have been shown to be an important tool to reduce malaria endemicity [16,17].

Along the Kenyan coast, there is no detailed information on the breeding sites of this species which is an important vector of malaria. Reported in this paper are the investigations on the determination of how the physicochemical and environmental factors in the larval habitats affect the survival, distribution and abundance of *An. merus*.

## Methods

### Study site

The study was carried out in Malindi district which is the tenth largest town in Kenya and a major tourist destination in Kilifi County along the Kenya Coast. Malindi town is approximately 108 km north of Mombasa. Entomological sampling was carried out in Garithe village located 27 kilometers north of Malindi town in Kenya. Garithe has been previously described [18]. The coastal part of Garithe consists of mangrove trees and the area experiences high tides every month leaving pools of water during the low tides. These pools of salty water provide suitable habitats for *An. merus* breeding. The area also has numerous pockets of man-made ponds [2].

### Larval habitat characterization

Characterization of a larval habitat required data from both environmental and physicochemical variables. The following environmental variables were recorded: Size of habitat (water depth, length and width), distance to nearest house, distance from the sea shore, canopy cover, algal cover, emergent plant cover, and turbidity. The physicochemical variables recorded were: salinity, dissolved oxygen, conductivity, water temperature, and pH.

For each larval habitat identified, the latitude and longitudinal co-ordinates were taken and recorded using a hand held Global positioning system (GPS) instrument (Garmin International Inc., Olathe, KS). For each habitat identified, the water depth, length and width were measured using a 1 M stick. Any measurements greater than 1 M were recorded as >0 1 M. Distance to the nearest house was measured using a tape. If distance measured were more than 100 M the distance was assessed visually and an estimate of the distance recorded. Canopy cover was measured as a percentage cover of shade over the habitat. Algal coverage was recorded as being present or absent. Emergent plants included both aquatic and immersed terrestrial vegetation and this was recorded as either present or absent. Turbidity was measured by placing the water in a clear glass container and placing it against a white background and recording it as either clear, low, medium or turbid.

The physicochemical variables were measured using a hand held field instrument, YSI 650 Multiparameter Display System (YSI environmental, YSI incorporated, Yellow springs Ohio USA). The machine automatically recorded pH, salinity, temperature, dissolved oxygen, total dissolved solids and conductivity. After the instrument was configured, the rod was placed in the water for one to two minutes after which it displayed the results of the readings on the screen and recorded.

### Larval sampling, storage and identification

Mosquito larvae were collected from the different aquatic habitats identified in Garithe. This was done in the morning hours between 0900 hrs and 1200 hrs. The habitats were first inspected for the presence of mosquito larvae. If mosquito larvae were present then 3–20 dips were taken with a standard mosquito dipper (350 ml) depending on the size of the habitat, this was done at each site. Mosquito larvae collected were kept in whirl packs and stored in a cool box. They were then transported to the laboratory in Malindi for further processing.

The third and fourth instar stage mosquito larvae collected were preserved in 100% ethanol, while the first and second stage instars were taken to the insectary at the Kenya Medical Research Institute (KEMRI) in Kilifi where they were reared using the water collected from their respective habitats and allowed to grow until they became 4^th^ instar larvae which were then identified and preserved in 100% ethanol. Each habitat was given a labeling code and all larvae collected were placed in different vials labeled according to the habitat they were collected from.

Pupae collected from the different habitats in the field were kept in emergent cages at the KEMRI insectary; the emerged adults were then identified as *Anopheles gambiae* morphologically using taxonomic keys of Gillies and De Meillon [19] and Gillies and Coetzee [20] using morphological identification [20] and preserved in ethanol for PCR. All *Anopheles gambiae s. l* identified were then further identified to sibling species using r-DNA polymerase chain reaction (PCR) analysis [21]*.*

### Data management and analysis

All data collected in the field was recorded in field sheets and note books. Simpson Diversity Index (D) was used to assess the species richness and abundance in Garithe study site in Malindi.

$$ D = 1-\left[\frac{\sum\ n\ \left(n-1\right)}{N\ \left(N-1\right)}\right] $$

Where D is Simpson diversity index, N is total number of species in community, n is proportion of each species.

Pearson’s correlation was used to evaluate how the physicochemical parameters associate with each other in the breeding habitat. Pearson’s correlation of coefficients was also done per habitat to determine if there was a positive association among the parameters measured with *An. merus* larvae. Larval abundance in relation to habitat diversity was done using (one way) analysis of variance and *t*-test was used to determine the significance statistically in the number of *Anopheles merus* larvae found in the habitats.

## Results

Six habitat types were found in Garithe, this included hoof prints, ponds, pools, road drains, swamp and wells. Amongst the habitats, ponds and pools were the most sampled habitats, while hoof prints, road drain, swamp and well were sampled less frequently.

From these habitats a total of 3,228 mosquito larvae and 347 pupae were collected in Garithe from September 2007 to March 2008. Of this, 53.7% (n = 1,732) were anophelines and 46.3% (n = 1,496) were culicines. Only about 67.15% (n = 1,163) of the anopheline larvae were early instars and 32.9% (n = 569) of the anopheline larvae were late instars. Using the F - distribution analysis of variance, there was a statistical significant difference in the number of early instar of *An. gambiae s.l* in all the habitats sampled (F _(5,117)_ = 2.404; P = 0.041), but in late instars of *An. gambiae s.l* there was no statistical significant difference in the number of late instars in all habitats sampled (F _(5,117)_ = 0.276; P = 0.926). All the late instars anophelines were morphologically identified as *Anopheles gambiae s.l.* The pupae collected were kept in emergent cages in the insectary where only 40.9% (n = 142) emerged as adults. Out of these, 21.12% (n = 30) emerged adults were identified as *Anopheles gambiae s.l* and were tested by r-DNA PCR to sibling species.

A high mean number of early and late instars of *Anopheles gambiae* were observed from ponds, pools and wells while low mean number of larvae was observed in road drain and swamp. High numbers of early instars were observed in hoof prints suggesting that they were the preferred oviposition sites for *An. gambiae*. More pupae and late instars were observed in ponds and pools but few were observed in hoof prints, suggesting a high survival rate in these habitats. No pupae were observed in road drains, swamps and wells suggesting that the larval production in these habitats was very low.

### Proportion, and distribution of *An. gambiae s. l* larvae found in Garithe

**Figure 1:**
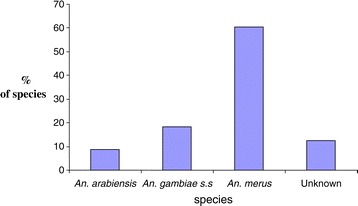
Proportion of Sibling species of *Anopheles gambiae s.l* larvae in Garithe.

A total of 159 morphologically identified late stage instar *Anopheles gambiae s.l* larvae were selected for r-DNA analysis by PCR. Out of these, 60.4% (n = 96) were *Anopheles merus,* 8.8% (n = 14) were *Anopheles arabiensis*, 18.2% (n = 29) were *Anopheles gambiae s.s* and 12.6% (n = 20) were unknown (Figure 1). The unknown samples tested PCR negative during the second and third PCR runs. This shows that *An. merus* larvae are the abundant species in Garithe.

**Figure 2:**
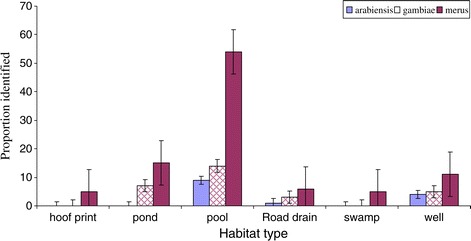
Distribution of *An. gambiae s.s, An. arabiensis, and An. merus* larvae species from the different habitats in Garithe.

The distribution of *An. gambiae s.s, An. arabiensis* and *An. merus* larvae from the different habitats in Garithe is shown in (Figure 2). Using paired *t*-test (t _(121)_ = −3.331, P = 0.001) a significantly high proportion of *An. merus* was observed in all habitats compared to *An. arabiensis*, and *An. gambiae s. s*.

*An. merus* was observed more in pools than in other larval habitat types. However, it was observed to occur in sympatry with *An. gambiae s.s* and *An. arabiensis* in ponds, road drains and wells. Swamps and hoof prints only supported the development of *An. merus* larvae as *An. gambiae s.s* and *An. arabiensis* were not observed in these habitats.

### Species diversity

Species richness and abundance was calculated within the *An. gambiae s. l* using the Simpson diversity index where D (diversity) was 0.582, this shows that there is moderate diversity of the species.

### Habitat characterization of *An. merus* in Garithe

Table 1 shows the means of the physicochemical and environmental parameters in different habitat types in Garithe. There was little variation in temperature (24.71 ± 0.28) and pH (8.47 ± 0.09) in all habitat types. There was significantly high salinity levels observed in ponds (57.06 ± 27.97), swamps (95.51 ± 79.27) and wells (69.95 ± 69.51) compared to pools (26.98 ± 11.71), road drain (0.38 ± 0.27) and hoof prints (42.70 ± 31.68). There was a wide range of salinity levels in swamps and hoof prints while in pools it varied slightly. Further, conductivity, dissolved oxygen and total dissolved solids (TDS) varied within and among the habitat types.

**Table 1:** **The means of the physicochemical and environmental parameters in the six different habitat types in Garithe**

**Habitat type**	**Temp (°C)**	**Conduct (S/m)**	**Salinity (dS/m)**	**Dissolved Oxygen (mgL)**	**pH**	**TDS (mg/L)**	**Can %**	**Algae %**	**Emerg %**	**Debris %**
Hoof print	23.94 ± 1.07^a^	53.62 ± 35.70^a^	42.70 ± 31.68^a^	170.14 ± 150.66	8.16 ± 0.25	39.68 ± 29.31^b^	0.00 ± 0.00	0.00 ± 0.00	0.00 ± 0.00	0.00 ± 0.00
Pond	24.18 ± 0.48^b^	120.76 ± 48.62	57.06 ± 27.97	140.79 ± 59.67	8.28 ± 0.13	262.58 ± 207.07^a^	22.61 ± 4.00	5.71 ± 2.21	4.38 ± 1.10	14.32 ± 2.36
Pool	25.12 ± 0.43	45.70 ± 15.43	26.98 ± 11.71	47.02 ± 33.76	8.64 ± 0.14	32.51 ± 11.32	16.32 ± 2.65	3.89 ± 1.47^a^	4.17 ± 1.01	12.96 ± 2.20
Road drain	24.56 ± 0.74	0.60 ± 0.56	0.38 ± 0.27	97.00 ± 48.63	8.51 ± 0.43	0.39 ± 0.35	14.00 ± 6.78	12.00 ± 12.00	0.00 ± 0.00	22.00 ± 8.60
Swamp	24.77 ± 0.77	13.49 ± 10.33	95.51 ± 79.27	95.71 ± 86.40	9.05 ± 0.46	70.61 ± 59.20	68.60 ± 15.81	16.00 ± 13.63	36.00 ± 22.04	20.00 ± 12.94
Well	25.24 ± 0.72	79.26 ± 61.49^b^	69.95 ± 69.51	21.12 ± 20.09	8.23 ± 0.45	50.95 ± 39.94^b^	10.91 ± 4.71^b^	5.45 ± 5.45	1.36 ± 0.97	13.64 ± 3.63
Total	24.71 ± 0.28	70.30 ± 18.49	40.28 ± 11.55	93.43 ± 26.87	8.47 ± 0.09	106.18 ± 66.03	19.19 ± 2.15	5.16 ± 1.24	4.77 ± 1.01	13.52 ± 1.45

^a^ = Pearson’s correlation is significant at the 0.01 level (2-tailed) with *An. merus* larvae.

^b^ = Pearson’s correlation is significant at the 0.05 level (2-tailed) with *An. merus* larvae.

**Key:** Temp = Temperature; Conduct = Conductivity; TDS = Total dissolved solids; Can = Canopy coverage; Emerg = Emergent plant coverage.

Using Pearson’s correlation analysis test, different parameters were found to be associated with the occurrence of *An. merus* larvae in the different habitats sampled. In hoof prints, temperature, conductivity, salinity and total dissolved solids were the key factors that determined presence of *An. merus*. In ponds, temperature and total dissolved solids determined the presence of *An. merus* while in pools algae was the only parameter associated with the presence of *An. merus* larvae. Interestingly, salinity in pools was not observed to be favourable to the development of *An. merus* larvae despite the favourable range of salinity levels. In wells, conductivity, TDS and canopy coverage influenced the presence of *An. merus* larvae; however, in road drain and swamp none of the physicochemical and environmental parameters were associated with the presence of *An. merus* larvae, even though *An. merus* larvae were sampled from these habitats.

The correlation coefficients among the chemical and environmental variables are shown in (Table 2). Using Pearson’s correlation of analysis 6 out of the 55 correlation coefficients (10.9%) were statistically significant, suggesting non-random association between some pairs of variables.

**Table 2:** **Pearson’s correlations (coefficient of correlations) between the various physicochemical parameters**

**Variables**	**Temp (°C)**	**Conduct (S/m)**	**Salinity (dS/m)**	**DO (mg/L)**	**pH**	**TDS (Mg/L)**	**Emerg %**	**Debris %**	**Canopy %**	**Algae %**
Cond	0.233*									
Sal	0.110	0.686**								
DomgL	-0.006	-0.124	-0.075							
PH	-0.103	0.028	-0.030	0.158						
TDS	0.173	0.115	0.070	-0.063	-0.126					
Emerg %	-0.048	-0.048	-0.039	0.019	0.031	-0.043				
Debris %	-0.099	-0.012	0.034	-0.104	-0.060	-0.031	0.104			
Can %	-0.142	0.083	-0.062	-0.173	0.095	-0.050	0.160*	0.088		
Algae %	0.117	0.017	-0.046	-0.022	-0.105	0.394**	-0.049	-0.015	-0.042	
*An. merus*	0.346**	0.189	0.264*	-0.200	-0.062	0.142	-0.096	-0.088	-0.029	0.151

**KEY:**

*Correlation is significant at the 0.05 level (2-tailed).

**Correlation is significant at the 0.01 level (2-tailed).

Temp = Temperature; Conduct = Conductivity; DO = Dissolved oxygen; TDS = Total dissolved solids; Can = Canopy coverage; Emerg = Emergent plant coverage.

A significantly high positive correlation of (34.6%) was observed between *An. merus* and temperature suggesting that this parameter is important for the development of *An. merus* larvae. *An. merus* also had a positive correlation with salinity of (26.4%), suggesting this species is able to tolerate the presence of salinity in its habitat. Conductivity also had a strong (68.6%) positive association with salinity, indicating that in this habitats conductivity as a factor made salinity to be available. Presence of algae in the habitats showed strong positive associations with TDS (39.4%), indicating that TDS as a factor encouraged the growth of algae.

## Discussion

In this study, in the different habitats, Anopheles and Culex species were collected but, the most abundant species was *An. merus*. This suggested that the area Garithe in Malindi district has favourable habitats for the survival and development of *An. merus* larvae. This is in conformity to previous findings [18,8], where large numbers of *An. merus* was observed in this area.

*An. merus* was observed to exist in sympatry with the other sibling species within the *An. gambiae s.l* complex namely *An. gambiae s.s* and *An. arabiensis*. The foregoing was also observed in earlier studies done in Garithe [8]. In addition, the same was reported in Madagascar where *An. merus* was always observed in sympatry with members of the *An. gambiae* complex [3].

The area Garithe is found near the sea shore and this meant that *An. merus* usually existed near the sea shore. The same was also noted in Madagascar where *An. merus* was sampled near the sea shore in the West coast of Madagascar [3]. Further observations indicating that *An. merus* was the most abundant species were also reported in Jimbo village along the Kenyan Coast [5]. But in contrast to this, in South Africa, some *An. merus* was sampled inland in saline pools [22]; this meant that *An. merus* could also be found inland, provided that the habitat in which they breed has conditions suitable for its development.

Each habitat in Garithe all had different physicochemical and environmental variables that were key determinants of the presence of *An. merus* larvae.

Temperature was a significant factor in the abundance of *An. merus* larvae in hoof prints and ponds. Previous studies have also found that moderately high temperatures were necessary for the optimum growth of Anopheles larvae; they further found that, high temperatures usually accelerated their growth [23]. Furthermore, other studies have observed that warm water also allowed more micro-organisms to grow, which provide food sources for mosquito larvae [10]. In addition, it was also noted that high temperatures support year round rapid decomposition of debris, leaf litter and dead algae, which provided food resources to the *Anopheles* larvae [9].

Salinity was also a significant factor in determining the abundance of *An. merus* larvae in hoof prints. High salinity levels were observed in swamps, which only *An. merus* larvae can survive in; in contrast to this, some *An. merus* were sampled in road drains which had low levels of salinity compared to the other habitats, this suggests that *An. merus* larvae can survive in a range of saline waters as was previously observed [5], it was found that, in their laboratory breeding experiments, *An. merus* larvae were capable of completing development between 0 - 100% saline water with an optimum development at 0%, 40% and 60% saline water [7].

Algae was a significant factor in the abundance of *An. merus* larvae in pools; this was also observed previously in Garithe, that algae favoured the abundance of *An. gambiae s.l* as it was the main source of its food [8].

Conductivity, total dissolved solids and canopy coverage was a significant factor in the abundance of *An. Merus* larvae in hoof prints and wells. In Banambani Mali, it was observed that conductivity and TDS have significant effects on niche partitioning of young *Anopheles* larvae [24] and this could be the reason for the survival of *An. merus* in wells and hoof prints in Garithe. Further, canopy coverage is known not to favour survival of the larvae of *An. gambiae* complex [25,26] where there was a significant negative correlation of the occurrence of *An. gambiae* larvae with canopy cover and emergent plants in natural habitats. It was interesting to note that in Garithe canopy coverage showed to be a significant factor with the occurrence of *An. merus* larvae in wells; the findings of [25,26] could be due to the fact that the studies were done in the Kenyan Highlands which is environmentally different from the Kenyan Coast.

## Conclusions

The *An. merus* larvae as they develop in their aquatic habitats are affected by the physicochemical and environmental parameters present in the habitats. Habitat types also play a role in influencing the abundance and survivorship of the larvae. The survivorship and abundance of larvae in turn affect the production of the adult *An. merus*, which may or may not transmit malaria.
